# The associations between common neuroimaging parameters of Progressive Supranuclear Palsy in magnetic resonance imaging and non-specific inflammatory factors – pilot study

**DOI:** 10.3389/fimmu.2024.1458713

**Published:** 2024-08-08

**Authors:** Piotr Alster, Dagmara Otto-Ślusarczyk, Michał Kutyłowski, Bartosz Migda, Alicja Wiercińska-Drapało, Joanna Jabłońska, Marta Struga, Natalia Madetko-Alster

**Affiliations:** ^1^ Department of Neurology, Medical University of Warsaw, Warsaw, Masovian, Poland; ^2^ Department of Biochemistry, Medical University of Warsaw, Warsaw, Masovian, Poland; ^3^ Department of Diagnostic Imaging, Mazowiecki Hospital Brodnowski, Warsaw, Poland; ^4^ Diagnostic Ultrasound Lab, Department of Pediatric Radiology, Medical University of Warsaw, Warsaw, Masovian, Poland; ^5^ Department of Infectious and Tropical Diseases and Hepatology, Medical University of Warsaw, Warsaw, Masovian, Poland

**Keywords:** neuroinflammation, neurodegeneration, PSP, atypical parkinsonisms, tauopathy

## Abstract

Progressive Supranuclear Palsy is an atypical parkinsonism based on tauopathic pathology. Growing interest is associated with the pathomechanism of this disease. Among theories analyzing this issue can be mentioned the one highlighting the significance of inflammation. In this study authors examined 14 patients with PSP-Richardson syndrome (PSP-RS) and 13 healthy volunteers using laboratory testing based on the analysis of interleukins 1 and 6 (IL-1 and IL-6), tau in the cerebrospinal fluid (CSF) and non-specific parameters of peripheral inflammation in the serum (IL-1, IL-6, neutrophils, lymphocytes, monocytes, platelets and the ratios based on the factors). All of the patients underwent neuroimaging using magnetic resonance imaging using 3 Tesla. The serum levels of IL-1 were positively correlated with the area of the mesencephalon, suggesting that higher levels of IL-1 are not linked with atrophic changes in this region, whereas serum levels IL-6 was positively correlated with frontal horn width and negatively correlated with superior cerebellar area. Additionally IL-6 in the serum was found to be correlated with neutrophil-to-high density lipoprotein ratio. The observations were not confirmed in the analysis of the levels of interleukins in the CSF. To the best of our knowledge this work is one of the first analyzing this issue. The outcome of the work shows that the role of interleukins associated with microglial activation may possibly differ in the context of neurodegenerative changes, moreover the role of peripheral inflammation in PSP requires further analysis.

## Introduction

Progressive Supranuclear Palsy (PSP) is a four repeats tauopathy in the group of atypical parkinsonism (1). Clinically it is defined as a syndrome associated with postural deficiencies, oculomotor dysfunction, akinesia and cognitive/language disorders (1). The disease was discovered in the seventh decade of the twentieth century, primarily it was defined as a single entity, since the release of the most contemporary criteria of diagnosis in 2017, it is presented as a group of clinical subtypes differing in the context of correlation of primary features (1, 2). The two major subtypes of PSP – Richardson’s syndrome (PSP-RS) and Parkinsonism Predominant (PSP-P) comprise up to 90% of cases (3). The main subtypes significantly differ in the context of progression, clinical manifestation and response to levodopa treatment (4). Though the descriptions of PSP subtypes seem more and more detailed, the pathophysiology of PSP is not fully explored. It is not verified whether certain mechanisms, commonly associated with PSP, as inflammation, oxidative stress, vascular abnormalities are a cause or consequence of these processes (5, 6). It is also not specified whether these pathways affect certain regions of interest in the central nervous system more severely (7). The inflammatory hypothesis regarding the pathogenesis of PSP described in contemporary literature does not clearly indicate whether the mechanism should be directly associated with clinical deterioration or whether its certain aspect provide a protective role in the course of neurodegeneration. The example of chronic traumatic encephalopathy due to its certain overlaps in context of tauopathic pathology may suggest that repeated stimulation causing inflammatory activation could be a factor in the pathogenesis of neurodegenerative diseases (8). The McGeer theory rccognized the chronic impact of microglial activation as an aspect of neurodegeneration (9). Nevertheless though neurodegenerative processes may present certain similarities, the studies performed on patients with parkinsonisms, revealed that generally the inflammatory activation is more pronounced in atypical parkinsonisms, when compared to Parkinson’s disease (PD) (10, 11). The data concerning this issue is not sufficiently explored due to the small number of researches evaluating inflammatory processes in atypical parkinsonisms and the lack of such works evaluating correlations between the inflammatory factors and atrophic changes in neuroimaging as in this study.

Based on previous examinations of this research group concerning the role of interleukins and neurotrophic factors in the analysis of PSP, it was revealed that the agents may have a significant in the differential diagnosis of two major subtypes of PSP – PSP Richardson’s syndrome (PSP-RS) and PSP-Parkinsonism Predominant (PSP-P) (12, 13). The goal of this work was to verify whether the processes associated with inflammation can be linked with the neurodegenerative changes in the disease manifested by atrophy in neuroimaging.

## Material and methodology

In this study 14 patients with PSP and 13 healthy volunteers aged 50-80 years old were recruited. All of the patients with PSP included in the study had possible or probable diagnosis of PSP according to the most contemporary Movement Disorders Society criteria of diagnosis released in 2017 (1). The deficits among patients were evaluated using the third part of Unified Parkinson’s Disease Rating Scale (UPDRS-III). The results of UPDRS-III of patients with PSP varied between 20 and 30. None of the patients received levodopa treatment or any drug associated with the impact on dopaminergic system. The duration of the disease among PSP patients varied from 3 to 6 years. Among exclusion criteria could be mentioned neoplasms, autoimmune disease, infectious diseases, dyslipidemia, other neurodegenerative disease, vascular changes in the central nervous system. Authors excluded from the study patients and healthy controls using drugs possibly impacting the inflammatory parameters, as well as using medications affecting the level of cholesterol (due to Neutrophil-to-High Density Lipoprotein Cholesterol evaluation) according to characteristics of medicinal product or literature were excluded from the study. The comorbidities were excluded due to their possible impact on the evaluated parameters in laboratory and neuroimaging examination. All of the participants included in the study were examined in the Department of Neurology and Department of Hepatology and Infectious and Tropical Diseases of the Medical University of Warsaw. The patients were examined by neurologists experienced in movement disorders. Each patient underwent blood collection, lumbar puncture and Magnetic Resonance Imaging (MRI). The MRIs were evaluated by a radiologist experienced in neuroimaging of parkinsonisms (14, 15).

### Blood and cerebrospinal fluid (CSF) collection

The blood samples were obtained from 14 patients with PSP, all of whom were hospitalized in the Department of Neurology at the Medical University of Warsaw. Blood samples and CSF (5 mL) were drawn into test tubes without anticoagulant and then centrifuged. The resulting serum and CSF samples were subsequently frozen at −80°C until analysis. The analysis involved determining the levels of IL-6 and IL-1β using commercial enzyme-linked immunosorbent assays (ELISAs), employing human (h) IL-6 HS and IL-1β ELISA kits from Diaclon SAS and Tau protein ELISA kit from Cloud-Clone Corp). Absorbance readings were measured at 450 nm using a plate reader, and marker concentrations were calculated based on standard curves.

All patients underwent comprehensive laboratory examinations, including blood morphology analysis, assessment of C-reactive protein (CRP) levels, and biochemical analysis, which included lipids profile and ferritin levels. None of the patients exhibited elevated infection markers, such as CRP or leukocytosis. Several ratios, including the neutrophil-to-lymphocyte ratio (NLR), lymphocyte-to-monocyte ratio (LMR), neutrophil-to-high density lipoprotein cholesterol (HDL-C) ratio (NHR), platelet-to-lymphocyte ratio (PLR), and neutrophil-to-monocyte ratio (NMR) were calculated. These ratios were determined by dividing the number of neutrophils by the number of lymphocytes, the number of lymphocytes by the number of monocytes, the number of neutrophils by the number of HDL-C, the number of platelets by the number of lymphocytes, and the number of neutrophils by the number of monocytes from the same blood sample, respectively. The initial assessment of neutrophil and lymphocyte counts was conducted utilizing the Sysmex XT 4000i automated hematology analyzer within the Department of Laboratory Diagnostics at the Mazovian Bródno Hospital in Warsaw.

### Magnetic resonance imaging

In the conducted study, all participants underwent magnetic resonance imaging (MRI) utilizing the Siemens 3.0T equipment, with subsequent assessment performed by a radiologist possessing over 5 years of expertise in neuroimaging, utilizing dedicated software (Syngovia Siemens) specialized software. Quantitative measurements were acquired from T2-weighted sequences, encompassing the evaluation of the pons (P) and midbrain (M) areas using freehand region of interest in the midsagittal plane, the average widths of the middle cerebellar peduncles (MCP) in the sagittal plane, the average widths of the superior cerebellar peduncles (SCP) in the coronal plane, the third ventricle based on three measurements (V3) in the axial plane at the level of anterior and posterior commissures, and the maximal width of the frontal horns of the lateral ventricles (FH). The Magnetic Resonance Parkinsonism Index (MRPI) was derived from the formula MRPI = (P/M) × (MCP/SCP), while the Magnetic Resonance Parkinsonism Index 2.0 (MRPI 2.0) was calculated as MRPI 2.0 = MRPI × (V3/FH), serving as quantitative measures for assessing parkinsonism based on structural ratios obtained from the MRI data.

### Statistical analysis

Data analysis were performed by using GraphPad Prism 8 (GraphPad Software, San Diego, CA, USA). The arithmetic means (X) along with their standard deviations (SDs) were calculated. A significance level of p<0.05 was deemed statistically significant, and appropriate statistical tests were employed for comparisons between means. All the data were tested normally distribution (normality estimation) of analyzed variables was assessed using the Shapiro–Wilk W test. Pearson’s correlation coefficients were employed to evaluate the significance of correlations between the various laboratory and neuroimaging markers. Due to the limited number of patients, lack of neuropathological verification and the singular evaluation, authors considered a sharpened threshold regarding correlations (r above 0.5 or below -0.5).

## Results

### Clinical characteristics of PSP patients and HCs

The clinical characteristics of the patients are comprehensively described in **Table 1**. This study included patients with PSP 14 (9 males and 5 females) and 13 HCs (5 males and 8 females). The average age of the patients with PSP was 70 ± 3.6 years, and that of the HCs was 50 ± 8.8 years. There were significant differences between patients with PSP and HCs in age (*p* < 0.0001).

**Table 1:** Characteristics of PSP patients and HCs.

	Healthy controls (n= 13)	PSP patients (n=14)	p
Sex (male:female)	5/8	9/5	
Age (years)	50 ± 8.8	70 ± 3.6	0.0001
Duration (years)		3-6	
WBC (x10^9^/L)	6.29 ± 3.9		
Neutrophils (x10^9^/L)	4.03 ± 3.06	4.36 ± 1.48	
Monocytes (x10^9^/L)		0.48 ± 0.17	
Lymohocytes (x10^9^/L)	1.25 ± 0.58	2.21 ± 1.30	0.0001
Plateles (x10^12^/L)	149 ± 90.1	208.7 ± 49.8	0.0001
RBC (x10^12^/L)	3.44 ± 0.68		
NLR	2.82 ± 1.89	1.85 ± 0.62	0.01
LMR		4.53± 1.14	
NHR	0.15 ± 0.19	0.10 ± 0.05	
PLR	92.5 ± 60	107.3 ± 37	0.05
NMR		9.28 ± 2.11	

WBC, white blood cells; RBC, red blood cells, NLR, neutrophil to lymphocyte ratio, LMR, lymphocye to monocyte ratio, NHR, neutrophil to high density lipoprotein ratio, PLR, platelet to lymphocyte ratio, NMR, neutrophile to monocyte ratio; the data shown are the means ± SD; a comparison of variables between PSP patients and HCs was performed by student’s t-test; p < 0.05 was considered to indicate statistical significance.

Also, there were substantial differences between the PSP patients and HCs in terms of the lymphocyte, platelets count, NLR and PLR (p < 0.05). Patients with PSP had substantially similar plasma neutrophiles counts but lower NLR than HCs (p < 0.01). In addition, the plasma platelets count, and PLR were significantly elevated in patients with PSP than in HCs (p < 0.0001, p<0,05 respectively).

### Pearson’s correlation analysis of the all variables in PSP patients

This study showed that the levels of IL-1 in the serum are positively correlated with the area of mesencephalon. The Pearson’s correlation coefficient between serum IL-1 levels and the area of the mesencephalon was found to be r=0.535208, p< 0.02. This positive correlation indicates that as IL-1 levels increase, there is no corresponding decrease in the mesencephalon area, implying a lack of detrimental impact on this region (**Table 2**). The concentration of IL-6 in is correlated positively with the frontal horn width (r=0.516973, p<0.02) and negatively and the area of superior cerebellar peduncle (r=-0.563613,p<0.01), which may suggest partial association with enhancement of neurodegenerative changes (**Table 2**). In the context of interleukins no other correlations were detected in the serum and CSF (**Tables 2**, **3**). Ferritin was found to be positively correlated with the levels of interleukin-1 (r=0,539486, p<0,02) and interleukin-6 (r=0,547016, p<0,02). IL-6 was found to be positively correlated with NHR (r=0,5229, p<0,02). The observations were not confirmed among healthy volunteers. Additionally, tau evaluation in the CSF, which was performed due to the lack of neuropathological verification, revealed significantly increased levels in the CSF when compared to control group. No significant correlation was detected in the context of tau concentration in the CSF and atrophic changes in MRI (**Table 4**).

**Table 2:** Correlation analysis of all variables in patients with PSP.

Variable	NLR		LMR		NHR		PLR		Neutrophiles		Monocyte		Lymphocytes		IL-6		IL-1b	
	r	*P*	r	*p*	r	*p*	r	*p*	r	*P*	r	*p*	r	*P*	r	*p*	r	*p*
III vent	0.072	0.40	0.326	0.12	0.07	0.39	0.267	0.17	0.072	0.40	-0.195	0.25	0.077	0.39	0.436	0.05	0.264	0.18
V3	0.137	0.32	0.370	0.19	0.146	0.30	0.416	0.05	0.137	0.32	-0.076	0.39	0.186	0.26	0.405	0.07	0.247	0.19
FH	0.02	0.47	0.08	0.78	0.021	0.47	-0.208	0.23	0.020	0.47	-0.310	0.14	-0.101	0.36	**0.516**	**0.02**	0.331	0.12
P	0.02	0.93	0.107	0.35	0.096	0.37	-0.285	0.16	0.022	0.46	-0.177	0.27	-0.09	0.37	-0.342	0.11	0.391	0.08
M	0.028	0.46	0.326	0.12	0.038	0.44	-0.235	0.20	0.028	0.46	-0.09	0.37	0.024	0.46	-0.082	0.39	**0.535**	**0.02**
MCP	0.497	0.03	0.272	0.17	0.169	0.28	0.295	0.15	0.497	0.03	0.263	0.18	0.273	0.17	-0.344	0.11	0.225	0.21
SCP	0.112	0.35	0.162	0.28	0.074	0.40	0.067	0.40	0.112	0.35	0.402	0.07	0.287	0.15	**-0.563**	**0.01**	-0.129	0.33
M/P	0.02	0.46	0.113	0.35	0.013	0.48	-0.150	0.30	0.022	0.46	-0.031	0.45	0.078	0.39	0.056	0.42	0.461	0.04
MRPI	0.08	0.38	-0.182	0.26	-0.043	0.44	0.197	0.24	0.087	0.38	-0.090	0.37	-0.108	0.35	-0.127	0.33	-0.311	0.13
MRPI 2.0	0.162	0.28	0.047	0.43	0.082	0.77	0.488	0.03	0.162	0.28	-0.014	0.48	0.05	0.42	-0.035	0.45	-0.184	0.26

These data were assessed based on the Pearson correlation test.

Pearson correlation was performed to evaluate the relationship between two continuous variables; p < 0.05 was considered to indicate statistical significance.

NLR, neutrophil to lymphocyte ratio; LMR, lymphocyte to monocyte ratio; NHR, neutrophil to high density lipoprotein ratio; PLR, platelet to lymphocyte ratio; V3; III vent, the third ventricle; FH, the lateral ventricles; P, pons; M, midbrain areas; MCP, the middle cerebellar peduncles; SCP, the superior cerebellar peduncles; MRPI, magnetic resonance parkinsonism index; MRPI 2.0, the magnetic resonance parkinsonism index 2.0.

Bolded - p values below 0.05 and r values below -0.5 or above 0.5.

**Table 3:** Correlation analysis between plasma IL-6, IL-1 beta.

	IL-6		IL-1 beta	
	r	p	r	P
Neutrophils (x10^9^/L)	0.379	0.08	0.435	0.05
Monocytes (x10^9^/L)	0.100	0.36	0.012	0.48
Lymohocytes (x10^9^/L)	0.398	0.07	0.398	0.14
Plateles (x10^12^/L)				
PLR	0.387	0.08	0.242	0.20
NLR	0.177	0.27	0.435	0.05
LMR	-0.238	0.20	0.347	0.11
NHR	**0.522**	**0.02**	0.387	0.08
NMR	0.027	0.46	0.295	0.15

Pearson correlation was performed to evaluate the relationship between two continuous variables; p < 0.05 was considered to indicate statistical significance.

PLR, platelet to lymphocyte ratio; NLR, neutrophil to lymphocyte ratio; LMR, lymphocyte to monocyte ratio; NHR, neutrophil to high density lipoprotein ratio NMR- neutrophil to monocyte ratio.

Bolded - p values below 0.05 and r values below -0.5 or above 0.5.

**Table 4:** Correlation analysis of CSF IL-6, IL1- beta and Tau with all variables in patients with PSP.

Variable	IL-6		IL-1 beta		Tau	
	r	*p*	r	*p*	r	*P*
III vent	0.020	0.47	0.319	0.13	0.07	0.39
V3	0.08	0.38	0.308	0.14	0.146	0.30
FH	- 0.02	0.46	0.04	0.44	0.021	0.47
P	- 0.278	0.46	0.266	0.17	0.096	0.37
M	0.113	0.34	0.309	0.14	0.038	0.44
MCP	-0.152	0.30	-0.438	0.06	0.169	0.28
SCP	0.087	0.38	-0.260	0.18	0.074	0.40
M/P	0.145	0.30	0.289	0.15	0.013	0.48
MRPI	-0.313	0.13	-0.341	0.11	-0.043	0.44
MRPI 2.0	-0.187	0.26	0.098	0.36	0.082	0.77

These data were assessed based on the Pearson correlation test. Pearson correlation was performed to evaluate the relationship between two continuous variables; p < 0.05 was considered to indicate statistical significance.

V3, III vent, the third ventricle; FH, the lateral ventricles; P, pons; M, midbrain areas; MCP, the middle cerebellar peduncles; SCP, the superior cerebellar peduncles; MRPI, magnetic resonance parkinsonism index; MRPI 2.0, magnetic resonance parkinsonism index 2.0.

## Discussion

The outcome of the study may suggest that inflammatory interleukins possibly play different roles depending on the regions of interest of the central nervous system. The unfavorable abnormalities within the SCP and FH, which were positively correlated with the level of IL-6, did not show any association with the levels of IL-1. On the other hand IL-1 was found to be positively correlated with the area of mesencephalon, which may suggest its role possibly counteracting neurodegeneration. Previous evaluations regarding the expression of IL-1 beta transcripts, revealed its increased levels in the substantia nigra (11). IL-1 beta and IL-6 as well as other cytokines secreted as a consequence of microglial activation were linked with neurodegeneration in atypical parkinsonisms (16). The increase of microglial derived cytokines was interpreted as a factor differentiating atypical parkinsonisms with PD (17).

The results obtained in this study may seem interesting in the context of staging of PSP based on MRI charts, which were indicated in a work by Planche et al. in 2024 (18). This work indicated major stages of progress in atrophic abnormalities. Among them could be mentioned: ventral diencephalon, pallidum, brainstem, striatum, amygdala, thalamus, frontal and occipital lobe (18). The outcome of this work and the discrepancies in the context of the impact of IL-1 and IL-6 on certain regions of interest may highlight the vulnerability to different factors depending on the stage. On the other hand previous studies performed by this research group on the possible role of IL-1 and IL-6 in the differential diagnosis of PSP subtypes – PSP-RS and PSP-P, showed that in the serum and cerebrospinal fluid the level of both interleukins was higher in the clinically more favorable subtype (PSP-P), than in PSP-RS which is affected by more rapid deterioration (13). A study by Nubling et al. indicated that though Cathepsin S is associated with the impact on tau oligomer formation through limited cleavage, the IL-6 serum levels correlated positively with disease severity in PSP (19). The outcome of the work by Nubling et al, combined with the results of this study, may suggest that IL-6 may have a role in the neurodegeneration of PSP. Additionally the fact that the level of IL-6 in the serum is positively correlated with NHR, may suggest that there is a possible link between peripheral inflammation and HDL (20). This seems possibly crucial in the context of previous studies regarding the permeability of brain-blood barrier in dyslipidemia and HDL abnormalities (21, 22). Both of the interleukins were found to be positively correlated with the concentration of ferritin. This fact combined by diversed links between the levels of interleukins in the serum and atrophic changes in MRI, highlights the limited feasibility of ferritin evaluation in the serum in the context of possible associations with commonly observed abnormalities in the neuroimaging of PSP.

The fact that no correlations were detected between the levels of interleukins in the CSF and atrophic changes evaluated in this study could be explained by the fact that the most significant impact of the inflammatory factors could be more pronounced in the initial year of the disease. In favor of this argument could be mentioned the results of the study regarding the assessment of interleukins in subtypes of PSP. In this the levels of both interleukins were higher in the serum and CSF among patients with PSP-P, the subtype linked with gradual deterioration and less pronounced atrophic changes of the brainstem. In this study the majority of patients were diagnosed with PSP-RS, in which the abnormalities in the context of levels of interleukins were found to be less deviated than in PSP-P in the previous study of this research group (13). Inflammatory factors may also play a role prior to initial symptoms of PSP, however these hypotheses cannot be verified using this study. Undoubtedly the role of certain interleukins associated with microglial activation may differ, this could be partly justified by the diversity of clinical manifestations in the initial years of PSP, which could likely be an effect of different profiles of cytokines affecting their pathomechanisms. Additionally possible evaluations of atrophic changes in other regions of interest associated with the pathogenesis of PSP e.g. pallidum, putamen, caudate, amygdala, supplementary motor cortex, thalamus, frontal pole, precentral gyrus, occipital fusiform gyrus could provide a broader perspective on the issue (17, 18, 23).

Due to the fact that the issue concerning inflammation in PSP is not widely explored, the work could not be sufficiently discussed in the context of consistencies and discrepancies in other studies. Apart from the study performed by this research group, which indicated interleukin-1 and interleukin-6 as factors possibly differentiating PSP-P and PSP-RS, there were several works evaluating inflammatory factors in PSP examination. The majority of these work were based on the examination of small numbers of patients. One of the studies showed the increase of expression of IL-1beta transcript in substantia nigra among patients with PSP (24). Other works revealed increased levels of interferon γ, IL-10, IL-18, IL-1β, IL-4, IL-6, transforming growth factor β1, and Tumor Necrosis Factor-α in PSP and Multiple System Atrophy when compared to PD (10). Evaluations concerning the possible significance of NLR on one hand showed significantly higher levels of PSP, when compared to PD, on the other no pronounced differences with Corticobasal Syndrome (CBS) were detected (11, 25). The possible modulation of microglial-derived factors may be interpreted as a target point in future therapies in neurodegenerative, however contemporarily the data on its significance in PSP is insufficient (26).

Authors are aware of the limitations of the study among which could be mentioned the limited number of patients, single evaluation and lack of neuropathological verification. The small number of patients is related to the rarity of the disease. Additionally the recruitment of patients with PSP is affected by pronounced cognitive and motor deterioration. Taking into account the fact that the median life expectancy of PSP-RS patients after diagnosis was estimated at the level of 5.6 years, the examination of patients with 3-6 years duration is on one hand initially affected by significant obstacles in recruitment, on the other the study though limited by its pilot character may provide information on the inflammatory profile of advanced stage patients (27). In this context obtaining a larger sample of advanced stage patients in a single center study is difficult. The generalizability of the results of this pilot study is undoubtfully affected by the limitations mentioned above, however due to its novelty, it can be interpreted as a point in further discussions, especially in exploring the pathogenesis and possible therapeutic binding factors. As the patients included in the study are alive, no neuropathological verification was performed, a verification of the increased level of tau among patients with PSP in the CSF due to the lack of neuropathological verification was performed. The work does not indicate subtypes of PSP. The assessment is based on the evaluation of non-specific inflammatory parameters. Moreover the imaging is based on selected regions of interest, which are most commonly evaluated in PSP. Due to the fact that authors intended to obtain a control group without significant comorbidities, based on the fact that the recruitment was partly performed during COVID-19 pandemic, authors were forced to obtain a control group significantly younger than the examined group, as the age-matched controls were excluded due to comorbidities possibly jeopardizing the results. Additionally some of the patients which could be age-matched with PSP patients were unwilling to perform examination during COVID-19 pandemic. Apart from the indicated limitations, this pilot study stresses an issue which is unexplored in contemporary literature.

## Conclusion

The work, though presenting the outcome of a pilot study, may suggest that the role of inflammatory factors in neurodegenerative diseases is not unequivocal. The possible significance of certain peripheral agents may depend on the type of inflammatory factor and the vulnerability of regions of interest affected by PSP. The evaluation of peripheral inflammatory factors should be interpreted as an initiatory point in further discussions concerning the course of neurodegeneration in tauopathic atypical parkinsonisms. Additional evaluation of the issue based on larger groups of patients is required.

## Data availability statement

The original contributions presented in the study are included in the article/supplementary material. Further inquiries can be directed to the corresponding author.

## Ethics statement

The studies involving humans were approved by the Bioethical Committee of Medical University of Warsaw—approval numbers: KB/139/2020 and KB/1243/2016. The studies were conducted in accordance with the local legislation and institutional requirements. The participants provided their written informed consent to participate in this study.

## Author contributions

PA: Conceptualization, Formal analysis, Funding acquisition, Investigation, Methodology, Project administration, Validation, Writing – original draft, Writing – review & editing. DO: Formal analysis, Investigation, Methodology, Software, Writing – original draft, Writing – review & editing. MK: Formal analysis, Investigation, Methodology, Software, Writing – original draft, Writing – review & editing. BM: Formal analysis, Investigation, Methodology, Software, Writing – original draft, Writing – review & editing. AW: Formal analysis, Investigation, Methodology, Software, Writing – original draft, Writing – review & editing. JJ: Formal analysis, Investigation, Methodology, Software, Writing – original draft, Writing – review & editing. MS: Data curation, Formal analysis, Investigation, Methodology, Resources, Software, Writing – original draft, Writing – review & editing. NM: Data curation, Formal analysis, Investigation, Methodology, Resources, Software, Writing – original draft, Writing – review & editing.
